# Genito-Urinary and Syphilitic Diseases

**Published:** 1900-10

**Authors:** Byron H. Daggett

**Affiliations:** Buffalo, N. Y.


### Progress in Medical Science.

### GENITO-URINARY AND SYPHILITIC DISEASES.

Conducted by BYRON H. DAGGETT, M. D., Buffalo, N. Y.

### NOTE ON THE FREQUENCY OF RENAL CASTS WITHOUT ALBUMINURIA.

MARTIN and JONES at a clinic at Victoria Hospital, Montreal, (*Philadelphia Med. Jour.*, September 8, 1900,) demonstrated that the presence of casts in urine without albumin is not uncommon. This condition is usually associated with acute poisoning, alcoholism, icterus, arteriosclerosis, pernicious fever and convalescence from acute nephritis.

Eichorst and Senator say that casts without albumin is very uncommon. Purdy states that he has never found casts without albumin at some period during the course of the malady. Various other observers note conditions in which casts are found without the presence of albumin. Von Jaksch notes the frequency of hyalin casts in urine otherwise normal. He states also that casts of any other variety are extremely uncommon without albumin. Martin and Jones tabulate a series of examinations made at the Royal Victoria Hospital, and allege that under all conditions the same specimen of urine which showed absence of albumin may frequently contain casts, hyaline, epithelial and granular. The subjoined statistical table "seems to illustrate the frequency with which casts may be found in the urine when albumin is absent.

"Under such conditions, then, it would seem difficult to estimate the true importance of casts in the urine, while it cannot but be believed that their occasional occurrence is devoid of the gravity in prognosis usually associated with their presence—*i.e.*, without other significant symptoms. This applies, at all events to the hyaline and granular varieties; the epithelial casts, on the other hand, being more uncommon, and doubtless of more serious import.

"Glancing over the conditions in which our few observations have been made, one is, perhaps, struck with the suggestion that irritating conditions are, after all, the main causes associated with the presence of renal casts—*i. e.*, just as bile acts as an irritant to the renal cells, so, too, would toxins, as they may occur in tuberculosis, pneumonia, typhoid fever, sepsis, etc., act similarly and render the cells of the kidneys incapable of performing their proper functions. Further observations will be necessary to give satisfactory evidence as to the more common causes of renal cast-formation. That their presence, however, without albumin, is more frequent than is usually believed, seems evident from the examinations of patients, as given above."

---

### PROSTATIC CALCULUS.

J. C. Spencer (*Philadelphia Med. Jour.*, September 8, 1900,) says prostatic calculi are divided into two classes: essential and adventitious or exotic. The former has its origin in the gland substance, the other originates outside of the glandular panenchyma. The composition of the essential differs chemically and morphologically from the adventitious form.

The essential variety was first observed by Morgagni, who likened these concretions to "small grains of snuff." Virchow made investigations regarding their nature in 1881. Iversein, in 1871, made a study of the nature and origin of these minute bodies. He says that the pigment in certain epithelial cells becomes freed from the cell-envelope and permeates the glandular parenchyma. These, then, become a nucleus about which concentric homogeneous layers form, of a substance which gives an amyloid reaction.

Stilling says that degenerated protoplasm of gland epithelium is the nidus of these concretions. About these granules colloid material forms, degenerates and responds to the iodin reaction. These granules are only found in adult life, that is, they do not form concretions in childhood or early manhood, as they are usually expelled through the acini and duct by muscular contraction, but result in the formation of a hyaline substance.

Posner, in 1889, said that this substance is the result of coagulation of fluid albumin or its saturation with lecithin.

Eastman and Hildebrand believe that amyloid matter is present in prostatic concretions.

It would seem fair to conclude from these observations that prostatic calculi originate in the protoplasm of degenerated gland epithelia about which colloid material collects and degenerates, then various mineral salts accrete. If uric acid, ammonio-magnesium, phosphate or calcium oxalate are present it indicates connection with the urinary passages, as these are urinary salts. Prostatic calculi vary in size from 1-200 of an inch to an orange. Large sizes result from accretion of urinary salts. The essential calculi are sphenoidal, smooth, hard and smaller in size.

The diagnosis depends to some extent upon the symptoms and size. The small ones make no symptoms, even those of good size often produce no subjective symptoms. The objective symptoms are hardness upon rectal palpation and urinary obstruction. If the calculus has eroded through into the urethra, it may be detected by the sound. If the calculi are multiple and in contact crepitus may be induced by palpation. Tuberculous nodules may be mistaken for calculi.

Virchow in his *Archives*, II., 9, describes the presence of calculi in the female prostate; the existence of such an organ has been described by Leukart. They are found in the acini and crypts, surrounded by epithelia; soft externally, internally more dense, brown in color and formed in layers.

Friedel found them in the gland of a man dead at 52 years of age. Cutting into the prostate he foun d grit, many crystals of phosphate of lime.

Paulicki found 240 calculi, size of grains of barley, in the prostate of a man 73 years old.

Edward Morris reports that he found five calculi, weighing five grams, in the middle lobe of the prostate.

G. Gross reports finding urethro-prostatic calculi, weighing 67 grams.

Van Thorlen removed a prostatic calculus, weighing forty giams, by cutting through the anterior rectal wall. This calculus had a uric acid nucleus, with a calcium carbonate envelope, hence it must have been of extraprostatic origin.

Hottute removed two small and one large (150 grams) calculi from a patient forty-five years of age. Their nuclei indicate extra-prostatic origin. During the past fifty years many others have reported cases of prostatic calculi, either essential or adventitious.

The abstractor removed a calculus from the prostate of a man 68 years old, at the Sisters Hospital, June, 1899. This calculus was imbedded apparently in the prostatic urticle. It was the shape, and the size of a lima bean, hard, light-gray in color, and smooth. He gave a history of several years of serious urinary disturbance. He had had a perineal section four years before without relief. Calculus was not diagnosticated until a perineal opening was made into the membranous urethra, when the stone was detected by the finger. It is comparatively within recent times that there has been authentic recognition of this condition.

---

BOTTINI'S OPERATION.

FREEMAN (*Denver Medical Times*, October, 1899,) sums up the advantages of the operation as follows :

1. There is no mutilation and no external wound, the manipulations being carried out through the urethra.

2. A general anesthetic, so dangerous in the old and debilitated, is not often necessary, local anesthesia being usually sufficient.

3. There is very little hemorrhage, the vessels being sealed by cauterisation.

4. There is comparatively small danger of serious infection and usually but moderate rise in temperature, the wounds being necessarily aseptic. The charred surfaces tend to prevent absorption until granulations appear.

5. In most instances patients may sit up and even walk about in a few days, which is of great advantage in those who are old and feeble.

6. The effects may be almost immediate, more or less urine being voided within a few hours, where it was previously impossible to pass a drop.

7. But few relapses have been observed; in fact, improvement has a tendency to be progressive.

8. The operation may be repeated, if for any reason the attempt has been unsatisfactory.

9. The mortality is lower than with other effective measures.

10. Patients will avail themselves of this method of treatment when they will refuse to submit to castration, prostatectomy, etc.

